# Calreticulin Blockade Attenuates Murine Acute Lung Injury by Inducing Polarization of M2 Subtype Macrophages

**DOI:** 10.3389/fimmu.2020.00011

**Published:** 2020-01-30

**Authors:** Zhilong Jiang, Zhihong Chen, Lu Hu, Lin Qiu, Lei Zhu

**Affiliations:** ^1^Department of Pulmonary Medicine, Fudan University Zhongshan Hospital, Shanghai, China; ^2^Shanghai Institute of Nutrition and Health, Chinese Academy of Sciences, Shanghai, China

**Keywords:** calreticulin (CALR), anti-calreticulin antibody (aCALR), macrophages, acute lung injury (ALI), cytokines

## Abstract

Calreticulin (CALR) has anti-tumor effects by increasing dendritic cell maturation and tumor antigen presentation. However, whether CALR affects macrophages and modulates progression of acute respiratory distress syndrome/acute lung injury (ARDS/ALI) remains unknown. In this study, we discovered that CALR protein was highly expressed in the mice with LPS-induced ALI and CALR expression level was positively correlated to the severity of ALI. Commercial anti-CALR antibody (aCALR) can neutralize recombinant CALR (rCALR) and suppress the expression of TNF-alpha and IL-6 in the rCALR-treated macrophages. Blocking CALR activity by intraperitoneal (i.p.) administration of aCALR significantly suppressed ALI, accompanied with lower total cell counts, neutrophil and T cell infiltration in bronchoalveolar lavage (BAL) and lung tissues. The expression of CXCL15, IL-6, IL-1beta, TNF-alpha, and CALR were significantly reduced, in association with more polarization of Siglec F+CD206+M2 subtype macrophages in the aCALR-treated mice. Pre-depletion of circulating monocytes did not abolish the aCALR-mediated suppression of ALI. Further analysis in bone marrow-derived macrophages (BMDMs) showed that aCALR suppressed the expression of CD80, IL-6, IL-1beta, IL-18, NLRP3, and p-p38 MAPK; but enhanced the expression of CD206 and IL-10. In addition, we observed more expression and phosphorylation of STAT6 in the aCALR-treated BMDM. Lack of STAT6 resulted in comparable and slightly higher expression of CALR, TNF-alpha and IL-6 in the aCALR-treated STAT6-/- BMDMs than the untreated cells. Therefore, we conclude that CALR is a novel biomarker in the evaluation of ALI. Blocking CALR activity by aCALR effectively suppressed ALI independent of circulating monocytes. Siglec F+CD206+M2 subtype macrophages and p38 MAPK/STAT6 signaling pathway played important role in the immune regulation of aCALR. Blocking CALR activity is a promising therapeutic approach in the treatment of ARDS/ALI.

## Introduction

Acute respiratory distress syndrome (ARDS) is a severe inflammatory lung disease with severe hypoxemia, diffuse bilateral pulmonary infiltrates with mortality rate up to 36–52% (1, 2). There is no effective therapeutics so far. Uncontrolled lung inflammation is a major reason for the high mortality rate (3). During initial phages, macrophages are activated and release a large amount of pro-inflammatory cytokines and mediators that effectively attract neutrophils and T lymphocytes into the lung tissues, contributing to the pathogenesis of ARDS and murine acute lung injury (ALI), an ARDS mouse model. Therefore, modulation of macrophage activation and polarization is an effective therapeutic approach in the treatment of ARDS/ALI. A body of evidences showed that macrophages can be modulated by many reagents (4–7). Our previous research revealed that antioxidant resveratrol can attenuate ALI through polarization of alternatively activated macrophage or M2 cells (8). The therapeutic effects were associated with STAT3/SOCS3 signaling pathway. Because STAT3 and SOCS3 forms a negative feedback loop in the lung inflammation of ALI, loss of SOCS3 can activate STAT3, induces more lung inflammation, and resulting in the resistant of ALI mouse model to resveratrol therapy (9, 10).

Recent study showed that calreticulin (CALR) is critically involved in anti-tumor activity in tumor animal models. CALR is a calcium-binding chaperone protein and located on the endoplasmic reticulum (11). A body of evidences showed that CALR is highly expressed in tumor cells and transported onto cell membrane to serve as a C1q receptor and prophagocytic signal for macrophage phagocytosis of cancer cells and dead cells (12, 13). Because the exposed CALR can be released into extracellular milieu (14, 15), the serum CALR is highly elevated in the tumor patients and positively correlated to the prognosis of tumor (16, 17). Therefore, serum CALR is considered as a useful biomarker in cancer diagnosis and prognosis evaluation. Because CALR can induce dendritic cell maturation, increasing antigen internalization and presentation, the biological function is beneficial for CALR in tumor growth control. A body of ligands were involved in facilitating CALR-mediated phagocytosis activity, such as C1q, CD91, mannose binding lectin (MBL) (18, 19), S-nitrosothiol (SNO)-surfactant protein D (SNO-SP-D) (20, 21), asialoglycans (22), lipoprotein receptor-related protein (LRP) (23), scavenger receptor A (SRA) (24), adiponectin and phosphatidylserine (25, 26). After interaction with these potential ligands, CALR is phosphorylated (27) and subsequently activates endocytic receptor protein CD91 or called alpha-2-macroglobulin receptor on macrophages, finally triggering some downstream signaling pathways, such as p38 MAPK (mitogen-activated protein kinase) and NF-kappaB (20, 28). It was reported that CALR-exposed dendritic cells had increased maturation status in the responses to living tumor cells, apoptotic cells and cell debris. Meanwhile, CD86, CD83, and CCR7 were up-regulated and accompanied with higher expression of pro-inflammatory cytokines, such as TNF-alpha, IL-6 (29). Thus, the CALR-activated dendritic cells have potent activity in priming anti-tumor cytotoxic CD8+ T lymphocytes (CTLs) and CALR is a potential molecular target in anti-tumor immunotherapy (30–32).

In addition to the phagocytotic effects of CALR in anti-tumor responses, CALR has also an important role in the pathogenesis of infectious and autoimmune diseases. CALR interacts with lipopolysaccharide (LPS), a component of the gram-negative bacterial cell wall via the N- and C-domain globular head region and the C-domain alone, participating in LPS-mediated anti-bacteria innate immunity (33, 34). In addition, CALR participated in pathogenetic inflammation through activation of integrin alpha subunits (ITGAs) and subsequently increasing adhesion and infiltration of both T cells and neutrophils (11). Previous studies showed that serum CALR was increased in the patients with rheumatoid arthritis (RA) and systemic lupus erythematosus (SLE) (24). However, the role of CALR in the development of RA and SLE is not well-investigated. Additional study *in vitro* proposed that recombinant oligomerized CALR can activate p38 MAPK/NF-kappaB signaling, increasing TNF-alpha and IL-6 expression in macrophages (24). However, contradictory to the pro-inflammatory role of CALR, recent reports also showed that CALR may have an anti-inflammatory function in other animal models. For example, Fischer et al. recently reported that recombinant human CALR can inhibit lipopolysaccharide (LPS)-induced inflammatory osteoclastogenesis in the mouse calvarial bone (35). Another report indicated that intraperitoneal injection of recombinant CALR fragment 39-272 (CRT/39-272) into animal model with experimental autoimmune encephalomyelitis (EAE) can significantly reduce the disease severity of EAE (36). CALR deficiency can increase the expression of pro-inflammatory cytokines and chemokines, such as IL-6 and monocyte chemotactic protein 1/CCL2 (MCP-1) in THP-1 macrophages (19). Therefore, CALR has a dual immunological role under different pathological condition and animal models. On the one hand, CALR activates macrophages by activation of CD91/p38 MAPK/NF-kappaB signaling pathway, subsequently inducing the production of pro-inflammatory cytokines. On the other hand, CALR suppresses inflammatory responses by increasing macrophage phagocytosis and clearance of dead cells. The beneficial effects of CALR are associated with increased inflammation resolution and damaged tissue repair (7, 37). However, it remains unknown whether CALR plays an important role in the progression of ARDS/ALI. Our results in this study showed that CALR expression level was highly elevated in mice with LPS-induced ALI and positively correlated with ALI severity and pro-inflammatory cytokine release. However, blocking CALR activity by anti-CALR antibody (aCALR) can effectively attenuate ALI. Pre-depletion of circulating monocytes did not abolish the suppressive effects. Siglec F+CD206+ M2 subtype macrophages and p38 MAPK/STAT6 signaling pathway were involved in the therapeutic process of aCALR. The study provided a novel therapeutic approach by targeting CALR signaling in the treatment of ARDS/ALI.

## Materials and Methods

### Mice and Treatment

Nine to twelve weeks old C57BL/6 male mice were given intraperitoneal (i.p.) injection with 14 μg/kg mouse anti-CALR antibody (aCALR) (Abcam, Cambridge, MA, catalog number: ab223614) and intratracheal (i.t.) injection with 5 mg/kg Lipopolysaccharides (LPS) from Escherichia coli O55:B5 (Sigma, St. Louis, Missouri, USA). The mice were injected with the same doses of goat IgG isotype and LPS were used as untreated control group. The mice were treated with PBS as naïve controls. Two days after treatment, bronchoalveolar lavage (BAL) and lung tissues were collected for analysis. All animals were housed and treated according to the guidelines of the Institutional Animal Care and Use Committee of the Fudan University, Zhongshan Hospital in China. All experiments were approved by the committee.

### ELISA Assay for Cytokines and CALR

TNF-alpha, IL-6, IL-1beta, IL-18, IL-10, and CXCL15 were measured by ELISA assay, according to industrial instructions (R&D systems). The ELISA kit for measurement of CALR protein level in cell supernatants, BAL, lung tissues, and blood was purchased from Signalway Antibody, LLC, College Park, Maryland, USA. Lung protein lysates were obtained by incubation of 30 mg lung tissues with 200 μl lysis buffer containing 50 mM Tris-HCl, pH 8.1, 1% SDS, 10 mM EDTA, 1 mM PMSF. The samples were sonicated for 10 s and kept on ice 20 min, then centrifuged at 12,000 rpm for 15 min.

### Flow Cytometry

0.3 × 10^6^ cell suspension from lung digests or BAL were incubated with antibody cocktail containing FITC-anti-CD206, PE-anti-CD45, PerC-Cy5-anti-F4/80, PE-Cy7-anti-Ly6G, APC-Cy7-anti-CD11b, BV421-anti-Siglec F (also known as CD170). All antibodies were purchased from BD Biosciences (Franklin Lakes, NJ), BioLegend and eBiosciences (San Diego, CA). The cells were stained with fluorescence-minus-one (FMO) antibody cocktail for negative staining control. After incubation with antibodies in PBS supplied with 3% FBS for 40 min at room temperature, the cells were then washed with PBS for 2 times and analyzed on BD FACSAria™ III instrument and BD FACSDiva™ software (BD Biosciences, San Jose, CA) within 48 h. All data was analyzed using FlowJo software, version 8.8.4 (Tree Star Inc.).

### Cell Immunostaining and CALR Binding Assay

Bone marrow was collected from hind limbs of naïve mice and bone marrow-derived macrophages (BMDMs) were obtained by maintaining bone marrow cells in RPMI1640 culture medium supplied with 10% fetal bovine serum (FBS) and 20% conditional media of NIH3T3 cells for 6 days. Then the BMDMs were treated with 1 μg/ml anti-CALR antibody (aCALR) (Abcam, Cambridge, MA, USA), 500 ng/ml LPS (Sigma), 40 ng/ml rTNF-alpha or 40 ng/ml rIL-6 (R&D systems) for 24 h. The untreated cells and LPS alone were used as controls. The expression of NLRP3 (NLR family pyrin domain containing 3), STAT6, total p38 MAPK and p-p38 MAPK in the cells were analyzed by immunostaining. Briefly, the treated cells were fixed with 4% PFA, blocked with 10% goat serum, and then incubated with rabbit primary antibodies against NLRP3 (Abcam), p38 MAPK, p-p38 MAPK (Cell signaling), and STAT6 (Bioss antibodies Inc., Woburn, MA, USA). All primary antibodies were 150-fold diluted in blocking buffer and incubated with the cells at 4°C overnight. Cy3-conjugated anti-rabbit IgG (Jackson ImmunoResearch, West Grove, PA, USA) was used as secondary antibody and incubated for 2 h. After extensive washing with PBS, nuclei were stained with blue 4′,6-diamidino-2-phenylindole (DAPI) for 3 min. For extracellular CALR binding assay, 2 μg/ml recombinant mouse CALR (rCALR-6×His, CUSABIO, Wuhan, China) was pre-mixed with different concentration of aCALR for 30 min at 37°C, then the mixture was added into BMDMs for 24 h. The cells incubated the same concentration of rCALR-6×His were used as controls. The rCALR binding activity on the treated cells was detected by addition of FITC-conjugated antibody against 6×His (Invitrogen). The positively stained cells (green) were visualized under fluorescence microscope.

### Statistical Analysis

Results are presented as the mean ± standard error (SE) of each group. All data were analyzed by One-Way ANOVA followed by Tukey's multiple comparisons or 2-tail unpaired student's *t*-test between two groups. A value of *p* < 0.05 was considered as statistically significant different.

## Results

### CALR Was Up-Regulated in a Mouse Model With LPS-Induced ALI

**Figure 1:**
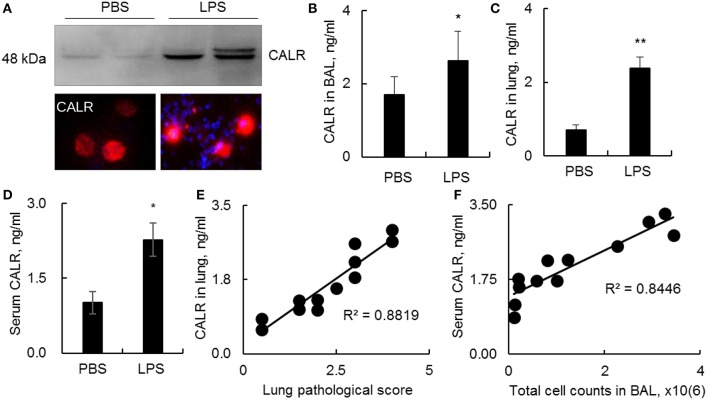
Calreticulin (CALR) was increased in a mouse model with LPS-induced ALI. C57BL/6 mice with ALI was established by intratracheal (i.t.) administration with 5 mg/kg lipopolysaccharides (LPS). BAL, serum and lung tissues were collected 2 days after the treatment. **(A)** CALR expression level in BAL was analyzed by Western blot analysis (upper panel). The expression in alveolar macrophages (AMs) was analyzed by immunostaining. The stained cells were visualized under fluorescence microscope (Magnification 400×). One representative photograph and Western blot of at least three independent experiments. The expression of CALR in BAL **(B)**, lung tissues **(C)**, and serum **(D)** was analyzed by ELISA analysis. Data was presented as mean ± standard error, *n* = 6. *p < 0.05, **p < 0.01 vs. PBS treated mice. **(E)** Correlation analysis between lung CALR protein level and lung pathological score. **(F)** Correlation between serum CALR level and total cell counts in BAL. Each dot represents individual sample from one mouse.

CALR is an intracellular calcium-binding chaperone protein, can be transported onto the outer face of the cell membrane and released into the surrounding milieu (14). However, it remains unknown whether CALR expression is affected during progression of ALI. In this study we analyzed CALR expression level in a mouse model with ALI. The results indicated that CALR was up-regulated in BAL as analyzed by Western blot, immunostaining and ELISA analysis (Figures 1A,B). The CALR was located in cytoplasm at low level, but mostly located onto cell membrane after cells were activated by LPS (Figure 1A, lower panel). In addition, we observed greater expression of CALR in lung digests and peripheral blood (Figures 1C,D). The increased CALR expression level was positively correlated to the disease severity and total cell counts in BAL (Figures 1E,F). Therefore, CALR would be a novel biomarker in the diagnosis and evaluation of ALI, and has a potential in clinical application. However, it is still unknown whether the altered CALR expression participates in the development of ALI.

### Anti-CALR Antibody Effectively Neutralized Recombinant CALR and Suppressed CALR Binding to BMDMs

**Figure 2:**
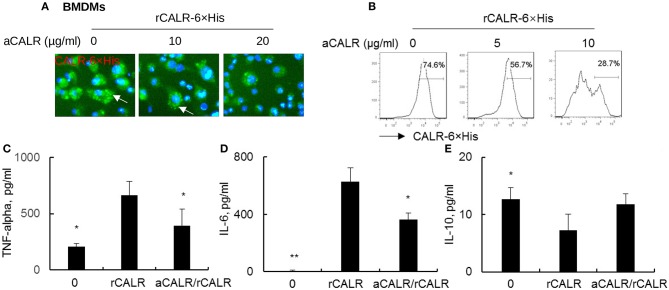
anti-CALR antibody (aCALR) inhibited recombinant CALR (rCALR) binding to macrophages and suppressed the expression of pro-inflammatory cytokines. Bone marrow-derived macrophages (BMDMs) were treated with 2 μg/ml rCALR-6×His with or without pre-neutralization with 5, 10, and 20 μg/ml aCALR. **(A)** Twenty-four hours after the treatment, rCALR-6×His binding to BMDMs was analyzed by incubation with FITC-conjugated anti-His antibody. White arrow indicates the punctuated positive stanning on cell surface. One representative photograph of three independent experiments. **(B)** rCALR-6×His positive cells were quantitatively analyzed by flow cytometry analysis. The expression level of TNF-alpha **(C)**, IL-6 **(D)**, and IL-10 **(E)** in the cell supernatants was analyzed by ELISA analysis. In the aCALR/rCALR group, rCALR was neutralized with 5 μg/ml aCALR **(C,D)** or 10 μg/ml aCALR **(E)**, respectively. Data was presented as mean ± standard error, *n* = 3. *p < 0.05, **p < 0.01 vs. rCALR-treated cells.

To confirm whether CALR is inflammatory and whether a commercial antibody against calreticulin (aCALR) can suppress extracellular calreticulin-induced inflammation of BMDMs, we incubated BMDMs with rCALR with or without neutralization by aCALR. Twenty-four hours after incubation, we observed that rCALR can effectively bind to the surface of BMDMs and be subsequently internalized into cytoplasm, as shown by punctuated positive staining of rCALR on the rCALR-treated cells (white arrow) (Figure 2A). In addition, neutralization with aCALR can effectively suppress rCALR binding to the cells, as shown by the reduced intensity of positive staining of the treated cells at aCALR concentration-dependent manner. Addition of 10 μg/ml aCALR approximately attenuated 50% rCALR binding activity as quantitatively analyzed by flow cytometry analysis (Figure 2B). The reduced rCALR binding activity was associated with lower expression levels of pro-inflammatory cytokines, such as TNF-alpha and IL-6 than the cells without pre-neutralization with 5 μg/ml aCALR (Figures 2C,D). However, we observed a trend of increased expression of IL-10 in the aCALR pre-neutralized cells (Figure 2E). Thereby, recombinant CALR had biological activity and can be effectively neutralized by aCALR. aCALR may be a potential therapeutic reagent in the treatment of inflammatory diseases, such as ARDS/ALI.

### Anti-CALR Antibody Suppressed Infiltration of Neutrophils and T Lymphocytes Into the Lungs of Mice With ALI

**Figure 3:**
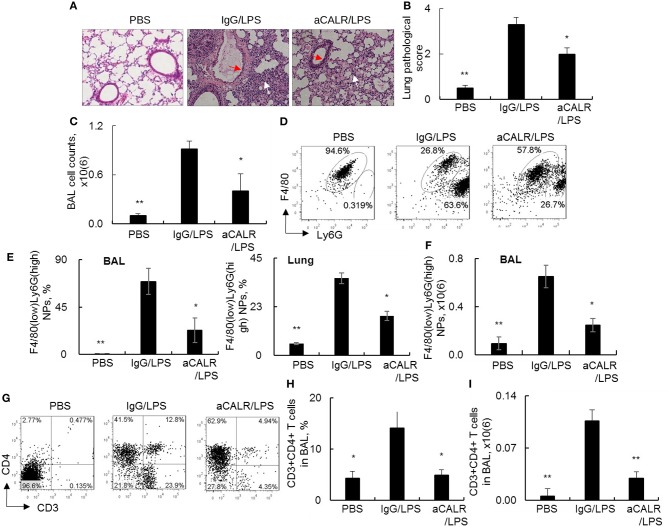
aCALR suppressed infiltration of neutrophils and T lymphocytes into the lungs of mice with ALI. C57BL/6 mice were intraperitoneal (i.p.) injected with 14 μg/kg aCALR, in conjunction with intratracheal (i.t.) injection with 5 mg/kg LPS (aCALR/LPS group). The mice injected with PBS alone (PBS group), IgG plus LPS (IgG/LPS group) were control groups. BAL and lung tissues were collected 2 days after treatment. **(A)** H&E staining for lung histology. Red arrow indicates epithelial cell hyperplasia and white arrow indicates inflammatory infiltrates. One representative photograph of each group. **(B)** Quantitative analysis of ALI severity by scale from 0 to 4 in terms of infiltrating inflammatory cells and alveoli destruction. **(C)** Total cell counts in BAL. **(D)** Flow cytometry analysis of neutrophils (NPs) and macrophages (MPs) in BAL. NPs were identified as F4/80(low)Ly6G(high) cells and MPs were identified as F4/80(high)Ly6G(low) cells. One representative dot plot of each group was shown. **(E)** Statistical analysis of the percentage of NPs in BAL (left panel) and lung digests (right panel). **(F)** Absolute number of NPs in BAL. **(G)** flow cytometry analysis for CD3+CD4+T lymphocytes in BAL. Quantitative analysis of the percentage **(H)** and absolute number **(I)** of CD3+CD4+ T cells in BAL. *n* = 5 mice/group for all quantitative analysis. Data was presented as mean ± standard error. *p < 0.05 and **p < 0.01 vs. IgG/LPS group.

To investigate whether blocking CALR activity by aCALR can affect the development of ALI, we intraperitoneal administered aCALR into the mice with LPS-induced ALI. The mice treated with the same doses of IgG isotype or PBS were used as controls. As a result, we observed the significant increased lung inflammatory infiltrates (white arrow) and epithelial cell hyperplasia (red arrow) in the lung of the ALI mouse model. However, the mice co-treated with aCALR significantly reduced the lung inflammation and epithelial cell hyperplasia (Figures 3A,B, *p* < 0.05). The reduced lung inflammation was accompanied with significantly lower total cell counts in BAL (Figure 3C). Further analysis by flow cytometry analysis indicated that aCALR reversed the LPS-induced increases in the percentage of F4/80(low)Ly6G(high) neutrophils (NPs), but the percentage of F4/80(high)Ly6G(low) macrophages (MPs) were relatively attenuated, due to massive influx of NPs into BAL (Figure 3D). The quantitative analysis showed that aCALR significantly decreased the percentage of NPs in BAL and lung tissues (Figure 3E). Accordingly, the absolute number of NPs in BAL was significantly reduced, as compared to the mice treated with IgG isotype controls (Figure 3F, *p* < 0.05). In addition to the suppressive effects on neutrophil infiltration, we also observed the significantly reduced percentage and absolute number of CD3+CD4+ T lymphocytes by aCALR treatment (Figures 3G–I), indicating the potent immune suppressive effects of aCALR *in vivo*.

### aCALR Attenuated the Expression of Pro-inflammatory Cytokines and CALR in Mice With ALI

**Figure 4:**
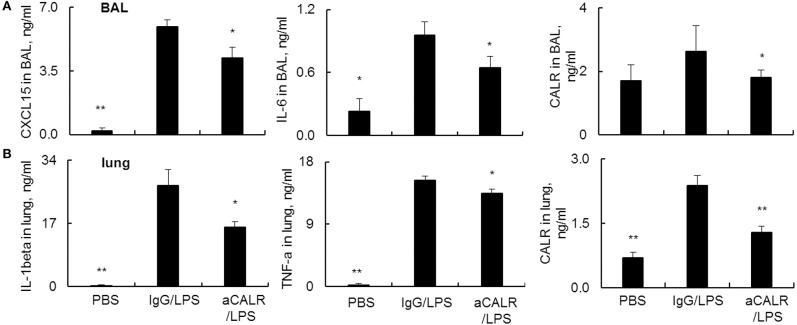
aCALR attenuated the expression of pro-inflammatory cytokines and CALR in BAL and lung tissue lysates of mice with ALI. ELISA analysis for CXCL15, IL-6, and CALR **(A)** in BAL, IL-1beta, TNF-alpha, and CALR **(B)** in lung tissue lysates. Samples were measured for three times and representative data of three independent experiments, mean ± standard error, *n* = 5 mice per each group. *p < 0.05 and **p < 0.01 vs. IgG/LPS group.

Pro-inflammatory cytokines are potent mediators in the pathogenesis of ALI. To address the suppressive effects of aCALR on ALI were mediated by the altered expression of cytokines in ALI, we analyzed the cytokine expression in BAL and lung protein lysates by ELISA assay. The results revealed that LPS significantly increased the expression of pro-inflammatory cytokines. However, aCALR treatment significantly reversed the LPS-induced up-regulation of CXCL15 and IL-6 in BAL (Figure 4A, *p* < 0.05). In addition, IL-1beta and TNF-alpha were also significantly downregulated in the lung protein lysates after aCALR treatment, compared to the control mice treated with IgG isotype (Figure 4B, *p* < 0.05). To define whether CALR expression was altered by aCALR treatment in ALI, we analyzed the CALR expression level by Sandwich ELISA assay, the results revealed that CALR expression level was increased in BAL and lung, but that was obviously attenuated by aCALR treatment (Figures 4A,B, right panel). Thereby, CALR expression was increased in ALI and may participate in the pathogenesis of ALI. Blocking CALR activity by aCALR should be a promising therapeutic approach in the treatment of ARDS/ALI.

### aCALR Suppressed ALI Independent of Circulating Monocytes and Modulated Macrophage Subtype Polarization

**Figure 5:**
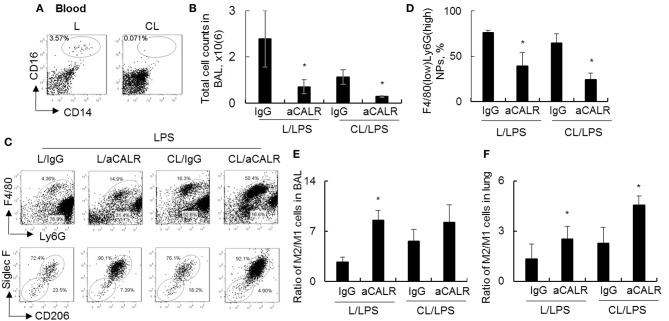
Pre-depletion of circulating monocytes increased M2 cell-biased polarization, but did not prevent aCALR-mediated suppression of murine ALI. Eight to ten weeks old male C57BL/6 mice were i.p. administered with 200 μl clodronate lyposome (CL) for depletion of circulating monocytes (MNs). The same doses of liposome (L) were used as control. **(A)** 2 days after the treatment, the population of MNs were analyzed by flow cytometry analysis. MNs were identified as CD14+CD16+ cells. One representative dot plot of three treated mice. Mice with or without MNs pre-depletion were i.t. injected with 5 mg/kg LPS in conjunction with i.p. injection with 14 μg/kg aCALR antibody (CL/aCALR/LPS or L/aCALR/LPS groups). The mice injected with goat IgG and LPS (IgG/LPS group) were control groups (CL/IgG/LPS or L/IgG/LPS groups). **(B)** Two days after the treatment, total cell counts in BAL were analyzed and data was presented as mean ± standard error. *p < 0.05 vs. IgG/LPS group, *n* = 3. **(C)** Flow cytometry analysis for infiltrating F4/80(low)Ly6G(high) neutrophils (NPs) and F4/80(high)Ly6G(low) macrophages (MPs) in BAL (upper panel). Macrophage subtypes were gated on MPs. M2 cells were identified as Siglec F+CD206+ cells; M1 cells were identified as Siglec F-CD206- cells (lower panel). Representative dot plot of individual mouse per group. **(D)** The percentage of infiltrating NPs in BAL was quantitatively analyzed. The ratio of M2/M1 cells in BAL **(E)** and lung tissues **(F)** was quantitatively analyzed. *p < 0.05 vs. IgG/LPS group. *n* = 3.

To investigate whether circulating monocytes (MNs) is necessary for aCALR-mediated immune suppression of ALI, we depleted MNs by clodronate liposome (CL) prior to aCALR treatment of ALI mice. The mice without pre-depletion of MNs were used as controls (L). Our results showed that 98% circulating CD14+CD16+ monocytes were depleted 2 days after CL administration (3.57–0.07%) (Figure 5A). In addition, MNs pre-depletion induced lower total cell counts and F4/80(low)Ly6G(high) neutrophils (NPs) in BAL (Figures 5B,C) and lung tissues (data not shown) of mice with ALI than the mice without MNs pre-depletion, the results were consistent with the results of our previously report (10). Furthermore, MNs pre-depletion did not abolish aCALR-mediated immune suppression of ALI, even additively reduced total cell counts and NPs influx into BAL (Figures 5B–D). Siglec F is a cell surface lectin that binds glycoconjugates containing sialic acids (38). Our additional study revealed a higher population of Siglec F+CD206+ M2 subtype macrophages, but lower population of Siglec F-CD206- M1 subtype macrophages in BAL (Figure 5C, lower panel). Quantitative analysis showed that population of Siglec F-CD206- M1 subtype macrophages were significantly reduced in aCALR co-treated mice with or without MNs pre-depletion (Supplemental Figure 1A). The similar effects were also observed in the lung tissues of aCALR co-treated mice; whereas the Siglec F+CD206+ M2 subtype macrophages were relatively increased (Supplemental Figure 1B). The biased polarization of macrophage subtypes induced higher ratio of M2/M1 cells in the BAL (Figure 5E) and lung tissues (Figure 5F) of aCALR-treated mice.

**Figure 6:**
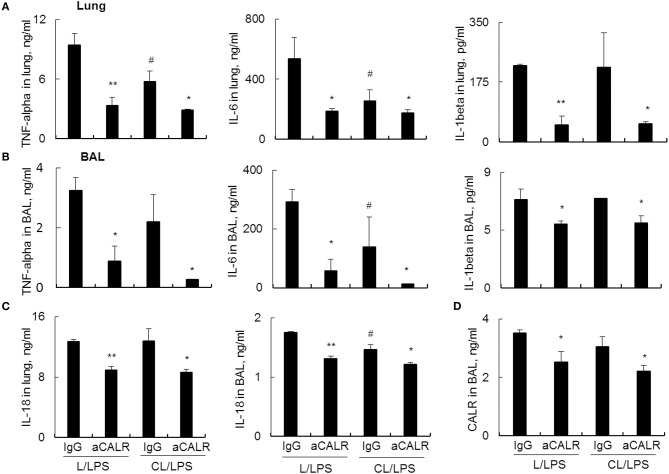
aCALR effectively suppressed the expression of cytokines and CALR in mice with ALI pre-depleted of MNs. ELISA assay for TNF-alpha, IL-6, and IL-1beta in the lung protein lysates **(A)** and BAL **(B)** of treated mice with or without pre-depletion of MNs. **(C)** ELISA analysis for the expression of IL-18 in lung and BAL. **(D)** Sandwich ELISA analysis for the expression of CALR in BAL. Samples were measured for three times and representative data of three independent experiments was shown. Data was presented as mean ± standard error. *p < 0.05, **p < 0.01 vs. IgG/LPS group, ^#^*p* < 0.05 vs. IgG/L/LPS group, *n* = 3.

Further analysis by ELISA assay showed that MNs pre-depletion in conjunction with administration of aCALR additively suppressed the expression of pro-inflammatory cytokines, including TNF-alpha, IL-6 and IL-1beta in lung tissues (Figure 6A) and BAL (Figure 6B). Cell pyroptosis is characterized with activation of NLRP3 inflammasome and release of both IL-1beta and IL-18 (39–41). Consistent with the results above, we observed moderately lower expression of IL-18 in the lung (Figure 6C, left panel) and BAL (Figure 6C, right panel) of aCALR-treated mice with or without MNs pre-depletion, indicating the suppressive effects of aCALR on macrophage pyroptosis independent of MNs. Similarly, MNs pre-depletion did not abolish the suppressive effects of aCALR on CALR expression in BAL (Figure 6D). Therefore, MNs are not necessary for aCALR-mediated immune suppression in murine ALI. aCALR may suppress acute lung injury and inflammation by targeting multiple cells, including alveolar macrophages (AMs) and lung epithelial cells, etc.

### aCALR Attenuated the Expression of Pro-inflammatory Cytokines in LPS-treated Bone Marrow-Derived Macrophages (BMDMs)

**Figure 7:**
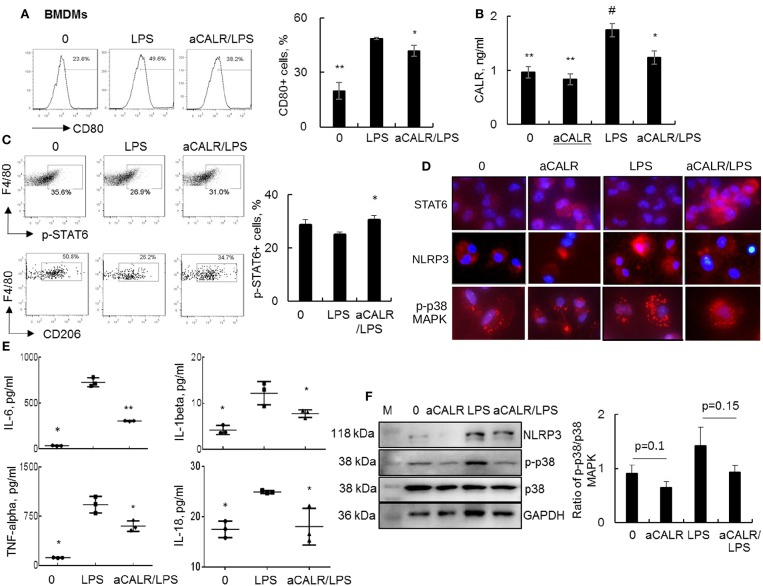
aCALR suppressed the expression of CALR and pro-inflammatory cytokines in LPS-treated BMDMs. BMDMs were treated with 500 ng/ml LPS with or without 30 min pre-treatment of 1 μg/ml aCALR. The cells untreated or treated with aCALR and LPS alone were controls. **(A)** Twenty-four hours after treatment, CD80 expression on F4/80+CD11b+ macrophages were quantitatively analyzed by flow cytometry analysis. The percentage of CD80+ macrophages was presented as histogram (left panel) and quantitatively analyzed (right panel). *p < 0.05, **p < 0.01 vs. LPS group, *n* = 3. **(B)** The expression level of CALR in supernatants of the treated cells was analyzed by Sandwich ELISA assay. ^#^*p* < 0.05, vs. the untreated cells; *p < 0.05, **p < 0.01 vs. LPS-treated cells, *n* = 3. **(C)** Flow cytometry analysis for p-STAT6 and CD206 on F4/80+CD11b+ macrophages. One representative data of three independent experiments. The percentage of p-STAT6+ cells were quantitatively analyzed (right panel). *p < 0.05 vs. LPS-treated cells, *n* = 3. **(D)** Immunostaining for the expression of STAT6, NLRP3, and p-p38 MAPK in BMDMs. Positively stained cells (red) were visualized under fluorescence microscope after incubation with Cy3-conjugated anti-rabbit IgG. Nuclei were stained with DAPI (blue). One representative photograph was shown for each treatment (magnification 400×). **(E)** ELISA analysis for IL-6, IL-1beta, TNF-alpha, and IL-18 in supernatants of the treated cells. *p < 0.05, **p < 0.01 vs. LPS-treated cells. **(F)** Western blot analysis for NLRP3, p-p38 MAPK and total p38 MAPK. Blots incubated with antibody against GAPDH were used as internal controls. M indicates protein marker and one representative blot of three independent experiments (left panel). Quantitative analysis of band intensity by Image J software (right panel). Data was presented as the ratio of target protein over internal control GAPDH and statistically analyzed by 2-tail student *t*-test.

To *in vitro* confirm whether aCALR exerted suppressive effect on ALI by targeting macrophages, we treated BMDMs with 1 μg/ml aCALR and 500 ng/ml LPS alone or both. The cells untreated or treated with aCALR and LPS alone were used as controls. Analysis by flow cytometry and ELISA indicated that the expression of CD80 and CALR was, respectively, increased on the LPS-treated cells and supernatants, but aCALR treatment reversed the LPS-induced up-regulation of CD80 and CALR (Figures 7A,B). Signal transducer and activator of transcription 6 (STAT6) is an important intracellular transcriptional protein member of JAK-STATs family. It was reported that STAT6 signaling promoted the expression of anti-inflammatory IL-10, increasing polarization of M2 subtype macrophages and inflammation resolution in some animal models (5, 42, 43). Our additional study by flow cytometry analysis showed that LPS treatment reduced the phosphorylation of STAT6 (p-STAT6) at residue pY641-STAT6 and the expression of CD206, a M2 cell surface biomarker on BMDMs was also suppressed by LPS. However, aCALR co-treatment reversed the LPS-induced down-regulation of p-STAT6 and CD206 (Figure 7C). The results were also confirmed by Western blot analysis (data not shown) and immunostaining, by which LPS moderately reduced the expression of STAT6, but aCALR co-treatment moderately enhanced the expression of STAT6 (Figure 7D). Thus, aCALR can induce M2 cell-biased polarization and STAT6 signaling may be involved in M2 cell-biased polarization after aCALR treatment.

To further define the biological function of aCALR-treated BMDMs, we further measured the expression levels of IL-6, IL-1beta, TNF-alpha, and IL-18 in cell supernatants by ELISA assay. We observed significantly more expression of IL-6, IL-1beta, TNF-alpha, and IL-18 in the LPS-treated cells, but that was significantly attenuated by aCALR co-treatment (Figure 7E, *p* < 0.05). Thereby, aCALR treatment increased M2 cell-biased polarization and the cells produced much lower pro-inflammatory cytokines.

IL-1beta and IL-18 are exclusively expressed in pyroptotic cells. NLRP3 is key protein component of NLRP3 inflammasome during cell pyroptosis (39, 44). To further define the role of aCALR on macrophage pyroptosis, we analyzed NLRP3 expression in the treated BMDMs. The results of immunostaining and Western blot analysis showed that aCALR suppressed the LPS-induced up-regulation of NLRP3. The results were consistent with a trend of reduced phosphorylation of p38 MAPK (p-p38) in the aCALR co-treated cells, compared to the cells treated with LPS alone (Figures 7D,F). Therefore, p38 MAPK/NLRP3 signaling maybe involved in the reduced macrophage pyroptosis after aCALR treatment.

### aCALR Attenuated the Up-regulation of CALR and NLRP3 in BMDMs After IL-6 and TNF-alpha Stimulation

We observed the increasing IL-6 and TNF-alpha in the mice and macrophages after LPS treatment, that was attenuated by aCALR treatment. However, whether aCALR may modulate macrophage function by blocking downstream signaling of IL-6 and TNF-alpha remains unknown. To address this issue, we stimulated BMDMs with recombinant IL-6 (rIL-6) and recombinant TNF-alpha (rTNF-alpha) with or without aCALR pre-treatment for 24 h. The expression of CALR in the treated cells were analyzed by flow cytometry and ELISA assay. Our results revealed that aCALR pre-treatment attenuated the expression of CALR, but increased the expression of p-STAT6 in the rIL-6 and rTNF-alpha-stimulated BMDMs (Supplemental Figures 2A,B). In addition, further analysis by immunostaining and Western blot analysis showed moderately reduced expression of NLRP3 and p-p38 MAPK in the aCALR pre-treated BMDMs after rIL-6 and rTNF-alpha stimulation (Supplemental Figures 2C,D). Quantitative analysis of Western blot analysis showed a trend of reduction in the ratio of p-p38/p38 MAPK and NLRP3/GAPDH in the aCALR pre-treated BMDMs (Supplemental Figure 2E). The results were consistent with the lower expression of pro-inflammatory cytokines, such as TNF-alpha, IL-6, and IL-1beta in the supernatants of the treated cells, but higher expression of anti-inflammatory IL-10 in the aCALR pre-treated BMDMs after rIL-6 and/or rTNF-alpha stimulation (Supplemental Figure 2F). Therefore, aCALR pre-treatment can modulate macrophage biological function not only stimulated by LPS, but also stimulated by rIL-6 and rTNF-alpha *in vitro*. aCALR may suppress macrophage activation by blocking downstream signaling of IL-6 and TNF-alpha.

### Lack of STAT6 Abolished aCALR-mediated Suppression of CALR, NLRP3, and Pro-inflammatory Cytokines in STAT6-/- BMDMs

**Figure 8:**
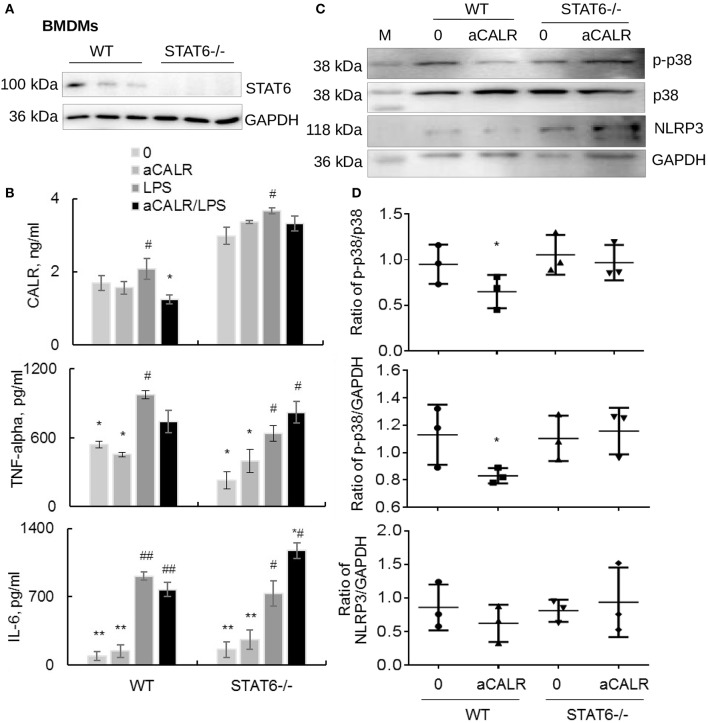
Lack of STAT6 abolished the immune suppressive effects of aCALR on STAT6-/- BMDMs. **(A)** The expression of STAT6 in BMDMs-derived from WT and STAT6-/- mice was analyzed by Western blot analysis. Each lane presents the cell lysates from individual mouse. **(B)** Sandwich ELISA analysis for the expression of CALR, TNF-alpha, and IL-6 in the supernatants of WT and STAT6-/- mice derived BMDMs. The cells were pre-treated with 1 μg/ml aCALR and then stimulated with 500 ng/ml LPS for 24 h. Data was presented as mean ± standard error, *p < 0.05, **p < 0.05 vs. LPS-treated cells, ^#^*p* < 0.05, ^##^*p* < 0.01 vs. the untreated cells, *n* = 3. **(C)** Western blot analysis for p-p38 MAPK, p38 MAPK, and NLRP3 in the treated cells. M indicates protein marker, one representative blot of three independent experiments. **(D)** The expression of p-p38 MAPK and NLRP3 was quantitatively analyzed. The data was presented as the ratio of p-p38/p38, p-p38/GAPDH, and NLRP3/GAPDH. Two-tailed Student *t*-test. *p < 0.05 vs. the aCALR-untreated cells.

Because aCALR can increase LPS-induced suppression of STAT6 *in vitro* (Figures 7C,D). To address whether STAT6 signaling is involved in the immune suppressive effects of aCALR in BMDMs, we cultured BMDMs isolated from STAT6-/- mice in RPMI1640 supplied with 10% FBS and 20% conditioned media of NIH3T3 cells for 6 days, then cells were treated with 500 ng/ml LPS for 24 h. The cells and supernatants were collected for analysis. The results of Western blot analysis confirmed the deficiency of STAT6 expression in STAT6-/- mice-derived BMDMs (Figure 8A). LPS moderately stimulated the expression of CALR in WT BMDMs, compared to the untreated controls. However, lack of STAT6 expression induced 2-fold more increases in CALR expression than that in WT BMDMs. In addition, aCALR pre-treatment moderately attenuated LPS-induced up-regulation of CALR in WT BMDMs, but not in STAT6-/- BMDMs (Figure 8B). The similar effects were also observed for the expression of TNF-alpha and IL-6, in which aCALR can moderately reduce the expression of TNF-alpha and IL-6 in WT BMDMs, but failed to attenuate the expression in STAT6-/- BMDMs. We even observed a trend of increasing expression of TNF-alpha and IL-6 in the aCALR-treated STAT6-/- cells compared to the untreated STAT6-/- cells (Figure 8B). The results indicated the involvement of STAT6 signaling in the aCALR-mediated immune suppression.

To further investigate the underlying molecular signaling pathway involved in the process, we analyzed the expression of p-p38 MAPK, p38 MAPK, and NLRP3 in the treated cells by Western blot analysis. The results revealed that co-treatment with aCALR can effectively suppress the expression of p-p38 MAPK and NLRP3, as shown by moderately reduced ratio of p-p38/p38, p-p38/GAPDH, and NLRP3/GAPDH in WT BMDMs. However, aCALR failed to suppress the expression of p-p38 MAPK and NLRP3 in STAT6-/- BMDMs (Figures 8C,D). Therefore, STAT6 signaling was critically involved in the aCALR-mediated suppression of macrophages.

## Discussion

Recent research has demonstrated the important role of CALR in anti-tumor activity by increasing dendritic cell maturation, tumor antigen presentation and priming of CD8+ cells (30, 45). CALR expression level is considered as an important indicator of tumor prognosis. Higher serum CALR level predicts higher survival rate and better antitumor responses during chemotherapy and immunotherapy. However, whether CALR is involved in the development of ARDS/ALI is not well-investigated so far. Our research in this study revealed that CALR expression level is a useful biomarker in the disease severity. There was a high level of CALR in BAL, lung tissues and serum of mice with ALI. The expression level of CALR was positively correlated to the disease severity of ALI. However, the studies about the role of CALR in ALI are still lacking. It was reported that soluble recombinant CALR (rCALR) polypeptides can increase production of TNF-alpha and IL-6, indicating the potent pro-inflammatory activity of CALR (24). The results were consistent with our *in vitro* study, by which rCALR up-regulated the expression of TNF-alpha, IL-6 and suppressed the expression of IL-10, but the effects were reversed by neutralizing rCALR with anti-CALR antibody (aCALR) *in vitro*. Therefore, CALR is pro-inflammatory and that can be neutralized by aCALR. Our further studies *in vivo* showed that intraperitoneal administration of mouse model with aCALR can significantly suppress ALI, in association with the reduced infiltration of neutrophils and T lymphocytes into the inflamed lung tissues, compared to the mice treated with LPS alone, indicating the pro-inflammatory role of CALR.

Our previous study showed that Siglec F+ subtype macrophages were decreased in the mice with ALI, but that was reversed by administration of anti-oxidant resveratrol, indicating a useful new biomarker of M2 macrophage, that was similar to CD206 (8). Consistent with the previous results, we in this study also observed significantly more population of anti-inflammatory Siglec F+CD206+ M2 subtype macrophages in the aCALR-treated mice with ALI. Supporting the anti-inflammatory function of Siglec F+CD206+ M2 subtype macrophages, the pro-inflammatory cytokines, such as IL-6, TNF-alpha, IL-1beta, IL-18, and CXCL15 were expressed at lower level in the BAL and lung tissues of aCALR-treated mice than the untreated mice. The results were consistent with the results *in vitro* study, by which the expression of IL-1beta, CXCL15, and IL-18 was suppressed in the aCALR pre-treated BMDMs than the untreated cells after LPS stimulation. The similar results were also observed in the TNF-alpha and IL-6 stimulated DMDMs, indicating the pivotal role of CALR signaling in the macrophage activation and polarization. TNF-alpha and IL-6 maybe immediate stimuli in up-regulation of CALR and subsequently increase pro-inflammatory responses of macrophages. Consistent with more anti-inflammatory Siglec F+CD206+ M2 subtype macrophages in the aCALR-treated mice, we also observed greater population of Siglec F+CD206+ M2 subtype macrophages in the aCALR-treated BMDMs. The role of Siglec F in promoting M2 subtype macrophages was supported by recent report, in which knockdown of Siglec-F can reduce M2 cell-specific STAT6 phosphorylation and expression of arginase-1 in IL-4 stimulated macrophages (38). Therefore, we conclude that CALR participated in the pathogenesis of ALI and aCALR may suppress ALI through increasing Siglec F+CD206+ M2 subtype macrophages.

To further investigate the role of circulating monocytes in the aCALR-mediated suppression of ALI, we pre-depleted circulating monocytes (MNs) by intraperitoneal injection of clodronate liposome (CL) before inducing ALI. We observed that aCALR can effectively suppress ALI in the mice pre-depleted of MNs, indicating that aCALR may suppress ALI independent of MNs. Other types of cells, such as AMs, lung epithelial and endothelial cells may be involved in the aCALR-mediated immune suppression. It warrants us to further investigate the role of CALR signaling in other cell types in the future.

NLRP3 is a key component of NLRP3 inflammasome during cell pyroptosis, contributing to the uncontrolled lung inflammation in ARDS/ALI (39, 41). However, it remains unknown whether macrophage pyroptosis is involved in the immune suppressive effects of aCALR. Our further analysis indicated that the expression of NLRP3 was moderately attenuated in the aCALR-treated BMDMs, compared to the cells-treated LPS alone. The suppressed NLRP3 inflammasome was correlated to lower expression levels of IL-1beta and IL-18 in the aCALR-treated cells. Therefore, we conclude that aCALR may suppress ALI through improving polarization of anti-inflammatory M2 subtype macrophages as well as suppressing macrophage pyroptosis.

It is previously reported that p38 MAPK participated in macrophage pyroptosis (41, 46). Our further analysis of p38 MAPK signaling indicated the involvement of p38 MAPK signaling in the aCALR-mediated immune suppression, because we observed moderately lower phosphorylation of p38 MAPK in the aCALR-treated BMDMs. We speculate that aCALR may suppress macrophage pyroptosis and subsequently reduced the release of IL-1beta and IL-18 through reducing p38 MAPK signaling pathway. However, recent reports revealed that the role of p38 MAPK signaling in modulation of macrophage biological function is controversial (5, 6, 42, 46). For example, p38 MAPK is considered as inducer of NF-kappaB and responsible for macrophage activation and pyroptosis (41). The dissembled SP-D and recombinant SP-A can activate NF-kappaB and increase the release of pro-inflammatory cytokines through increasing CALR/CD91/p38 MAPK signaling pathway (28, 47, 48). However, p38 MAPK is also considered as M2 cell inducer (5). For example, it was reported that docosahexaenoic acid (HA) can promote macrophage polarization toward CD206+ M2 subtype through p38 MAPK/STAT6/PI3K-AKT signaling pathway in macrophages, because inhibition of p38 MAPK resulted in downregulation of CD206 in docosahexaenoic acid (DHA)-treated cells (6, 49). In addition, the anti-inflammatory effect of p38 MAPK was associated with increasing expression of IL-10 (42, 43). Thus, we speculate that aCALR suppressed macrophage pyroptosis possibly through reducing p38 MAPK signaling pathway. More investigation is required to define the role of p38 MAPK/STAT6 signaling pathway in aCALR-modulated macrophage pyroptosis.

In addition to the involvement of p38 MAPK signaling, we also observed the involvement of STAT6 signaling in aCALR-mediated immune suppression. Jyk2/Jak1/STAT6 signaling pathway is considered important in inducing M2 subtype macrophages after stimulation with IL-4 and IL-13 (50, 51). Our study *in vitro* showed that the expression and activation of STAT6 were suppressed in LPS-treated macrophages and but increased by aCALR co-treatment *in vitro*. It would be helpful to define whether IL-4 and IL-13 are up-regulated in the aCALR-treated macrophages and mice, and further determine the role of IL-4 and IL-13 in the up-regulation of STAT6 after aCALR treatment in the future. To further investigate the role of STAT6 in aCALR-mediated immune suppression, we treated STAT6-/- derived BMDMs with LPS alone or conjunction with aCALR. As a result, we observed comparable expression level of soluble CALR in the supernatants of STAT6-/- derived BMDMs with or without aCALR pre-treatment. We even observed relatively more expression of TNF-alpha and IL-6 in the aCALR pre-treated STAT6-/- cells than the untreated STAT6-/- cells after LPS stimulation. The comparable expression of these cytokines was also observed in the STAT6-/- cells stimulated with TNF-alpha and IL-6 (data not shown). The results further confirmed the role of STAT6 signaling in aCALR-mediated macrophage function after stimulation with LPS as well as TNF-alpha and IL-6. According to the previous report that p38 MAPK is upstream of STAT6, promoting phosphorylation of STAT6 (5). We conclude that aCALR suppressed macrophage activation possibly through modulation of p38 MAPK/STAT6 signaling pathway. aCALR promoted M2 cell-biased polarization and suppress macrophage pyroptosis through suppressing the expression and activation of p38 MAPK and NLRP3, subsequently increasing the expression and phosphorylation of STAT6.

**Figure 9:**
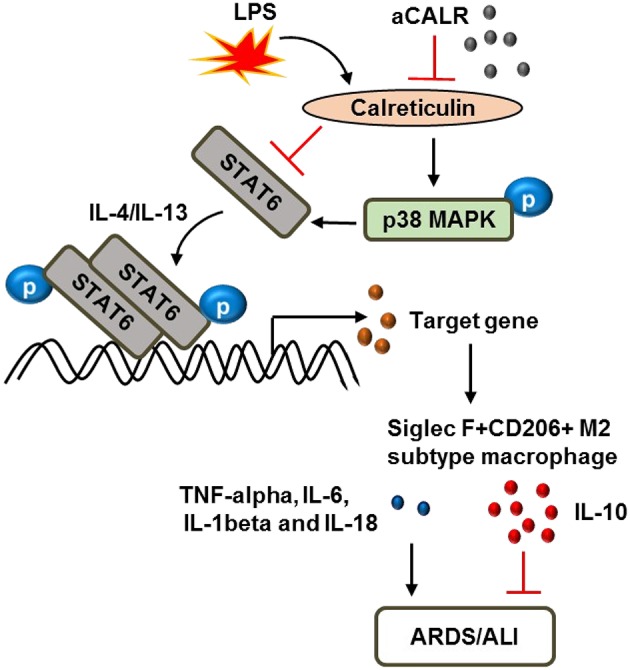
Schematic diagram of calreticulin (CALR) downstream signaling pathway in the modulation of macrophages. LPS up-regulates CALR and subsequently suppresses the expression and phosphorylation of STAT6, but enhances the phosphorylation of p38 MAPK. The improved STAT6/p38 MAPK signaling pathway increases polarization of Siglec F-CD206- M1 subtype macrophages and promoting ARDS/ALI through increasing expression of pro-inflammatory cytokines, such as TNF-alpha, IL-6, IL-1beta, and IL-18, etc. Anti-calreticulin antibody (aCALR) up-regulates the expression of STAT6 and pY641-STAT6, but reduces the activation of p38 MAPK by neutralizing CALR binding activity to macrophages. The suppressed STAT6/p38 MAPK signaling pathway promotes polarization of Siglec F+CD206+ M2 subtype macrophages, causing lower expression of pro-inflammatory cytokines, but higher expression of anti-inflammatory cytokines, such as IL-10, ultimately inhibits ARDS/ALI.

Taken together, we conclude that CALR is a novel biomarker in the evaluation of ARDS/ALI. CALR is pro-inflammatory in the development of ALI/ATDS. Blocking CALR bioactivity by aCALR suppressed LPS-induced development of murine ALI. The protective effects were independent of MNs and associated with more polarization of Siglec F+CD206+ M2 subtype macrophage and lower macrophage pyroptosis. The p38 MAPK/STAT6 signaling pathway was involved in the protective effects of aCALR, in association with the suppressed expression of pro-inflammatory cytokines and but increased production of anti-inflammatory cytokines from macrophages (Figure 9). Thereby, CALR is a potential therapeutic target in the treatment of ARDS/ALI.

## Data Availability Statement

The datasets generated for this study are available on request to the corresponding author.

## Ethics Statement

The animal study was reviewed and approved by the Institutional Animal Care and Use Committee of the Fudan University, Zhongshan Hospital, China.

## Author Contributions

ZJ participated in the generation of hypothesis, animal and cell experiments, data analysis and assembly, manuscript writing, and revision. ZC and LH participated in Western blot analysis. LQ provided technical assistance for flow cytometry. LZ participated in the generation of hypothesis. All authors read and approved the final manuscript.

### Conflict of Interest

The authors declare that the research was conducted in the absence of any commercial or financial relationships that could be construed as a potential conflict of interest.

## Supplementary Material

Supplemental Figure 1 Pre-depletion of circulating monocytes did not abolish the effects of aCALR on M2 cell-biased polarization in BAL and lung tissues of mice with ALI. Flow cytometry analysis for subtypes of infiltrating F4/80(high)Ly6G(low) macrophages (MPs) in BAL **(A)** and lung tissues **(B)** of the treated mice with ALI. The Siglec F-CD206- M1 cells and Siglec F+CD206+ M2 cells were quantitatively analyzed and data was presented as the percentage of positive macrophages. ^*^*p* < 0.05 vs. IgG/LPS group. *n* = 3.

Supplemental Figure 2 aCALR downregulated the expression of CALR and pro-inflammatory cytokines in IL-6 and TNF-alpha treated BMDMs. BMDMs were treated with 40 ng/ml IL-6, 40 ng/ml TNF-alpha, with or without pre-treatment with 1 μg/ml aCALR. **(A)** Twenty-four hours after treatment, the expression levels of CALR and p-STAT6 were analyzed by flow cytometry analysis. The cells were gated on F4/80+CD11b+ macrophages. Data was presented as histogram. One representative data of three independent experiments. **(B)** Quantitative analysis of CALR in the supernatants of the treated cells by ELISA assay. Two-tailed Student *t*-test, *n* = 3. **(C)** Immunostaining for the expression of NLRP3 in the treated cells. The positive cells were stained with red in cytoplasm (magnification 400×). **(D)** Western blot analysis for NLRP3, p-p38 MAPK and p38 MAPK in the treated cells. M indicates protein marker, one representative blot of three independent experiments. **(E)** The expression of p-p38 MAPK and NLRP3 was quantitatively analyzed. The data was presented as the ratio of p-p38/p38 and NLRP3/GAPDH. **(F)** The expression of TNF-alpha, IL-6, IL-1beta, and IL-10 in the supernatants of treated cells were measured by ELISA assay. ^*^*p* < 0.05, ^**^*p* < 0.01 vs. the cells untreated with aCALR. *n* = 3.
