# Supplementary figures and images for: Calreticulin Blockade Attenuates Murine Acute Lung Injury by Inducing Polarization of M2 Subtype Macrophages
Source: Front Immunol. 2020 Jan 30;11:11. doi: 10.3389/fimmu.2020.00011 (PMC7002388; doi:10.3389/fimmu.2020.00011)

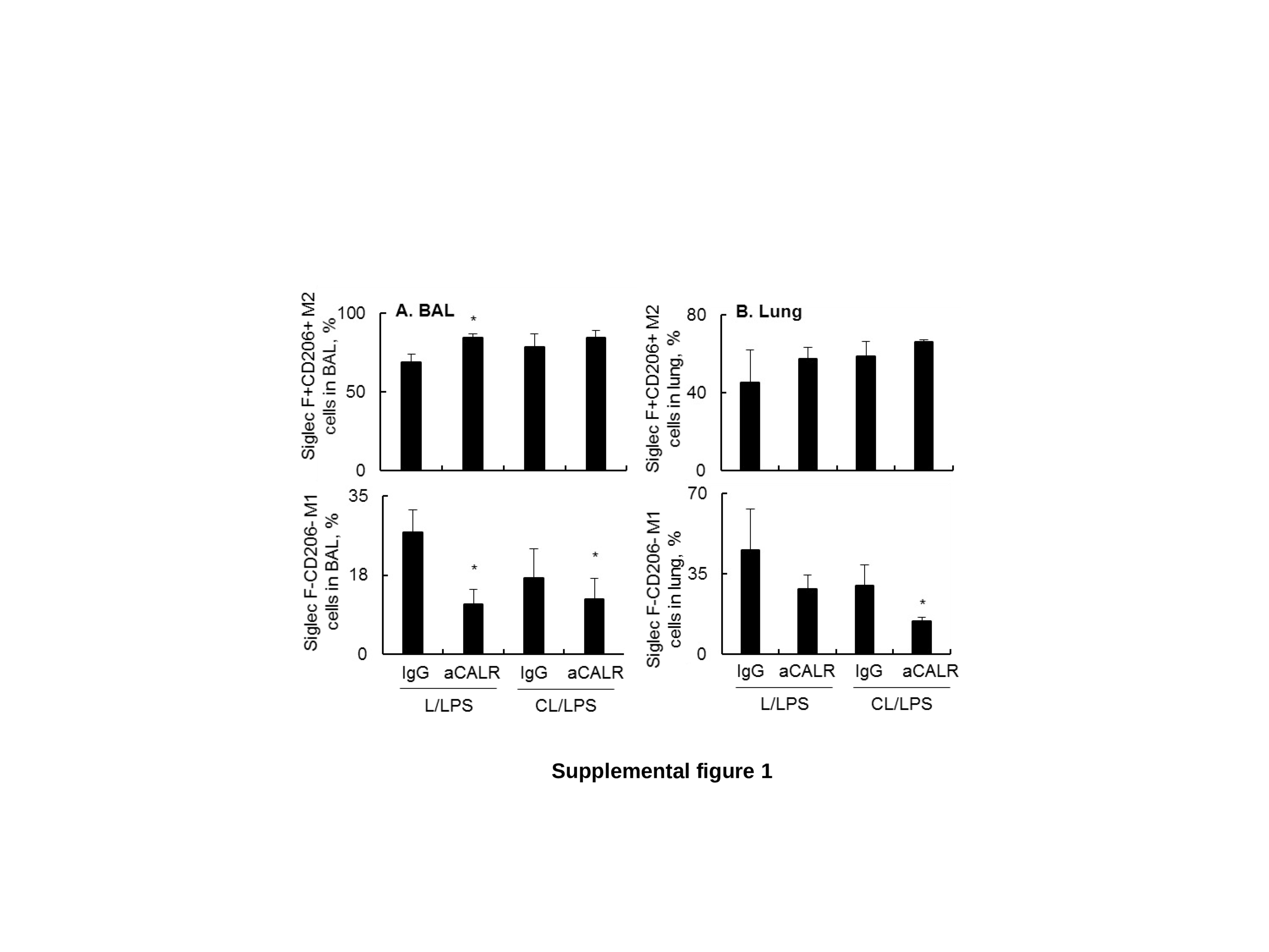

Supplement: Supplemental Figure 1 — Pre-depletion of circulating monocytes did not abolish the effects of aCALR on M2 cell-biased polarization in BAL and lung tissues of mice with ALI. Flow cytometry analysis for subtypes of infiltrating F4/80(high)Ly6G(low) macrophages (MPs) in BAL (A) and lung tissues (B) of the treated mice with ALI. The Siglec F-CD206- M1 cells and Siglec F+CD206+ M2 cells were quantitatively analyzed and data was presented as the percentage of positive macrophages. *p < 0.05 vs. IgG/LPS group. n = 3. [file Image_1.JPEG]

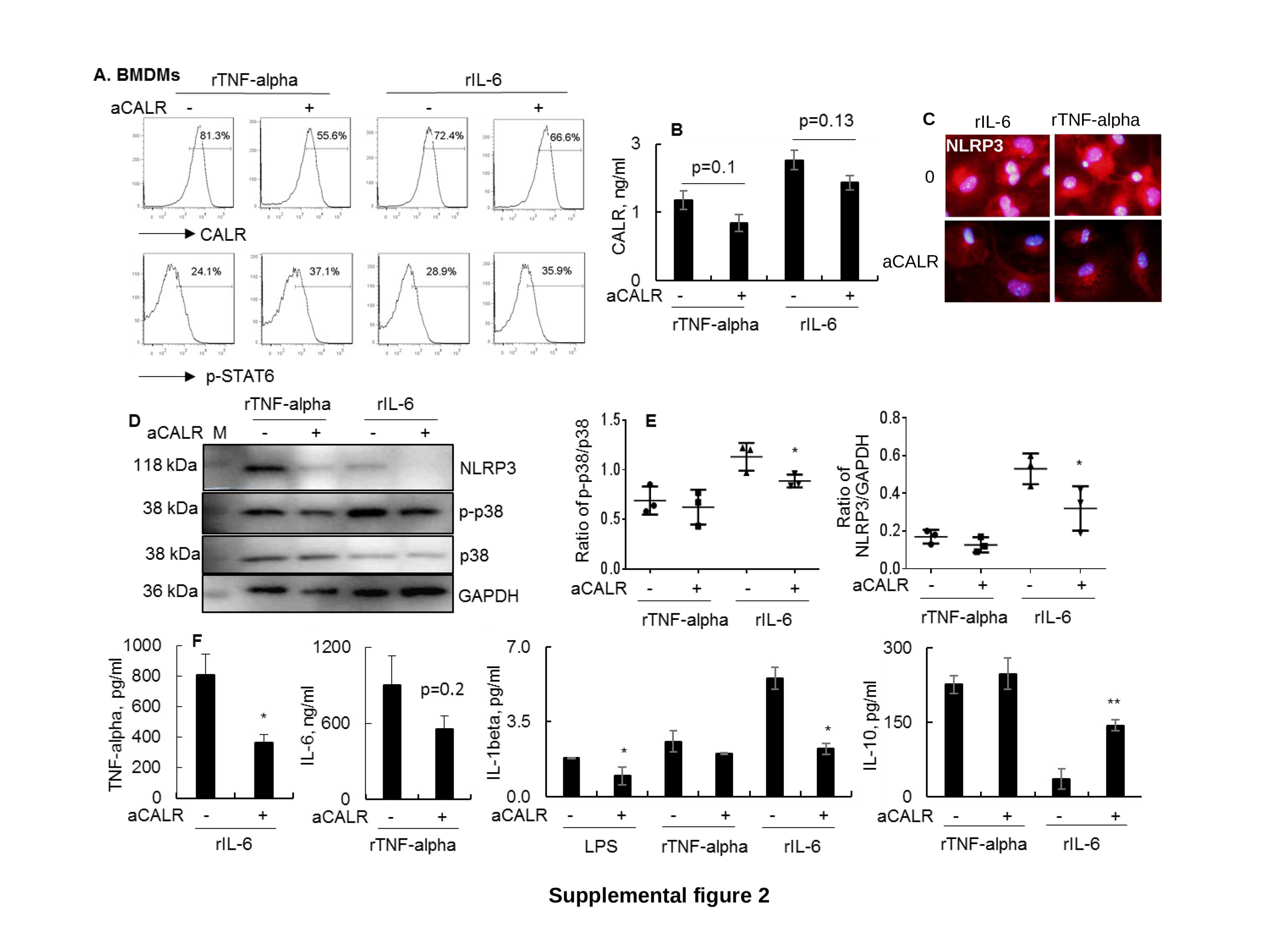

Supplement: Supplemental Figure 2 — aCALR downregulated the expression of CALR and pro-inflammatory cytokines in IL-6 and TNF-alpha treated BMDMs. BMDMs were treated with 40 ng/ml IL-6, 40 ng/ml TNF-alpha, with or without pre-treatment with 1 μg/ml aCALR. (A) Twenty-four hours after treatment, the expression levels of CALR and p-STAT6 were analyzed by flow cytometry analysis. The cells were gated on F4/80+CD11b+ macrophages. Data was presented as histogram. One representative data of three independent experiments. (B) Quantitative analysis of CALR in the supernatants of the treated cells by ELISA assay. Two-tailed Student t-test, n = 3. (C) Immunostaining for the expression of NLRP3 in the treated cells. The positive cells were stained with red in cytoplasm (magnification 400×). (D) Western blot analysis for NLRP3, p-p38 MAPK and p38 MAPK in the treated cells. M indicates protein marker, one representative blot of three independent experiments. (E) The expression of p-p38 MAPK and NLRP3 was quantitatively analyzed. The data was presented as the ratio of p-p38/p38 and NLRP3/GAPDH. (F) The expression of TNF-alpha, IL-6, IL-1beta, and IL-10 in the supernatants of treated cells were measured by ELISA assay. *p < 0.05, **p < 0.01 vs. the cells untreated with aCALR. n = 3. [file Image_2.JPEG]
